# Enzymes from Marine Polar Regions and Their Biotechnological Applications

**DOI:** 10.3390/md17100544

**Published:** 2019-09-23

**Authors:** Stefano Bruno, Daniela Coppola, Guido di Prisco, Daniela Giordano, Cinzia Verde

**Affiliations:** 1 Department of Food and Drug, University of Parma, Parco Area delle Scienze 23A, 43124 Parma, Italy; stefano.bruno@unipr.it; 2 Institute of Biosciences and BioResources (IBBR), CNR, Via Pietro Castellino 111, 80131 Napoli, Italy; daniela.coppola@ibbr.cnr.it (D.C.); guido.diprisco@ibbr.cnr.it (G.d.P.); daniela.giordano@ibbr.cnr.it (D.G.); 3 Department of Marine Biotechnology, Stazione Zoologica Anton Dohrn, Villa Comunale, 80121 Napoli, Italy

**Keywords:** Arctic/Antarctic environment, biocatalysis, cold-adaptation, marine biotechnology

## Abstract

The microorganisms that evolved at low temperatures express cold-adapted enzymes endowed with unique catalytic properties in comparison to their mesophilic homologues, i.e., higher catalytic efficiency, improved flexibility, and lower thermal stability. Cold environments are therefore an attractive research area for the discovery of enzymes to be used for investigational and industrial applications in which such properties are desirable. In this work, we will review the literature on cold-adapted enzymes specifically focusing on those discovered in the bioprospecting of polar marine environments, so far largely neglected because of their limited accessibility. We will discuss their existing or proposed biotechnological applications within the framework of the more general applications of cold-adapted enzymes.

## 1. Introduction

The economic progress in the “Era of Biotech” demands new biocatalysts for a wide range of applications, from therapy to industrial manufacturing. Particularly, biocatalysis is an attractive alternative to chemical synthesis, as biocatalysts are biodegradable and non-toxic, they originate from renewable sources, they have high selectivity and they provide products in high yields [1]. Up to 40% of the industrially relevant chemical reactions that require organic solvents harmful to the environment could be substituted by enzymatic catalysis by 2030 [2]. Many industrial processes already benefit from the use of enzymes (e.g., proteases, amylases, cellulases, carboxymethylcellulases, xylanases) in the production of pharmaceuticals, foods, beverages, confectionery, paper, as well as in textile and leather processing and waste-water treatment. Most of these enzymes are microbial in origin because they are relatively more thermostable than their counterparts from plants and animals [3]. Their market is expected to reach $7.0 billion by 2023 from $5.5 billion in 2018, with an annual growth rate of 4.9% for the period 2018–2023 [4]. In this market, industrial detergents will reach $10.8 billion by 2022, with an annual growth rate of 4.2% for the period 2017–2022 [4], whereas the global market for enzymes in the food and beverages industries is expected to grow from $1.8 billion in 2017 to nearly $2.2 billion by 2022, with an annual growth rate of 4.6% from 2017 to 2022 [4].

The increasing demand for new or better biocatalysts with different substrate selectivity, chiral selectivity, stability and activity at various pH and temperatures is likely to be met through the investigation of microorganisms, the largest reservoir of biological activities in Nature. Marine microorganisms are particularly promising in this respect and it is predicted [5] that the global market for marine biotechnology, estimated at $4.1 billion in 2015, may reach $4.8 billion by 2020 and $6.4 billion by 2025, with the identification of new marine-derived enzymes [6]. The bioprospecting of marine environments, i.e., the systematic search for new bioactive compounds or biomolecules, has been traditionally focused on temperate/tropical latitudes [7], and polar marine environments have been mostly neglected, mainly due to their limited accessibility. These habitats include not only seawater but also sediments and sea ice, where internal fluids remain liquid in winter. Although both polar regions contain freezing seas, their microorganisms have different evolutionary histories due to their different geography and land-sea distribution [8]. The northern polar region is characterized by extensive, shallow shelves of landmasses that surround a partially land-locked ocean [9], whereas the Antarctic region consists in a dynamic open ocean that surrounds the continent [10,11]. The opening of the Drake Passage between Tierra del Fuego and the Antarctic Peninsula 23.5–32.5 million years ago was the key event for the development of the Antarctic Circumpolar Current (ACC), partially responsible for cooling of the Antarctic waters. The Antarctic Polar Front, the northern boundary of the ACC, promoted the isolation of the Antarctic marine fauna [10,11]. In both polar regions, the constantly low temperatures have driven the evolution of cold-adapted microorganisms, which are classified as psychrophilic and psychrotolerant according to the physiological features of their growth [12]. In this review, the general term “cold-adapted” will be used to indicate “psychrophile” organisms indigenous to cold environments [13].

In view of recent breakthroughs in sampling methodologies, sequencing, and bioinformatics, polar marine environments have recently come into the scientific spotlight—also because of growing concerns about their role in global climate change dynamics. An increasing number of works demonstrated their biological diversity [14,15,16,17,18,19], which includes bacteria, archaea, yeasts, fungi and algae [20,21]. Among polar microorganisms, those defined as ‘cold-adapted’ are those that thrive in permanently cold environments, even at subzero temperatures in super-cooled liquid water, and that evolved the physiological and biochemical capability to survive and reproduce under these extreme conditions [22]. With a better knowledge of marine microorganisms of polar regions, previously inaccessible bio-products, both in terms of new bioactive metabolites and proteins/enzymes with potential commercial applications, have been described [23,24]. Their biotechnological use can take many forms, from agricultural production to industrial processes, food chemistry [25], synthetic biology, and biomedical uses [26,27]. It is expected that, in the near future, more cold-adapted bacteria and their enzymes will find their way to biotechnological applications.

In this review, we will describe recent findings in enzymes isolated in either Arctic or Antarctic regions, some of which are of potential biotechnological interest. Unlike previous reviews, we particularly focused on enzymes isolated from marine polar environments. Cold-adapted enzymes from non-marine environments (i.e., from soil or lakes) or from non-polar sources (i.e., deep sea) will be reported to suggest potential future applications for enzymes isolated from marine polar microorganisms or to highlight structure-function relationships common to all cold-adapted enzymes, regardless of their origin. 

## 2. Methods for Enzyme Discovery and Engineering

**Figure 1:**
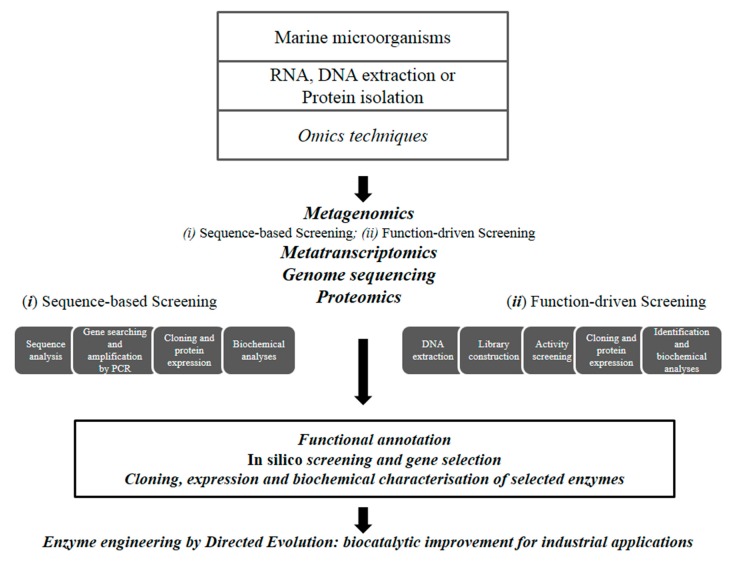
Workflow for the discovery of novel industrial enzymes by *omics* technologies.

The identification of enzymes from cultured or uncultured marine microorganisms have been carried out through several ‘-omics’ techniques. Additionally, natural enzymes have been optimized through enzyme engineering to improve their biotechnological properties (Figure 1).

*Omics techniques.* The overall knowledge on polar microorganisms has recently increased through the application of “omic” approaches (environmental shotgun sequencing, metatranscriptomics, proteomics) that have revealed the peculiar properties of their communities [28].

High-throughput metagenomic screening approaches, using both sequence-based and function-driven screenings, are contributing to the identification of a large number of enzymes, with most industrial enzymes having being identified by traditional functional screening of (meta)genomic libraries [29,30,31]. Recently, rapid technological developments in bioinformatics have revolutionized the exploration of the microbial diversity for the discovery of enzymes with commercial impact [32]. The sequence-based analysis of their (meta)genomic DNA by high-throughput sequencing methods followed by in silico analysis was used to predict enzyme function [33] allowing an increase in the rate of discovery. Sequence-based metagenomic approaches represents a powerful tool by avoiding the intensive, expensive, and time-consuming lab work associated to classic screening by reducing the number of targets to be functionally tested. Nevertheless, the latter approach is only limited to the discovery of genes with high similarities to already deposited sequences, making the discovery of new enzymatic functions difficult [34]. 

Some recent EU FP7-funded projects (e.g., MACUMBA and PHARMASEA) made valuable efforts in exploiting the potential of microorganisms (mostly bacteria and microalgae) for pharmaceutical, cosmeceutical, and nutraceutical applications [35]. In order to explore marine biodiversity in several environments, including extreme habitats, a pan-European consortium was instituted in 2015 [36] for the creation of one of the largest collections of marine enzymes, with a focus on those of interest for the industrial market [32]. At present, this collection contains about 1000 enzymes, of which around 94% are available in ready-to-use expression systems and 32% have been completely characterized [2,32,37]. However, the publication of gene/genome sequences is faster than the identification of new protein functions, the most important challenge in modern biology due to the lack of experimental evidence to support functional annotation of a large fraction of genes and proteins [38,39]. Indeed, there is a gap between the numbers of biological activities predicted in silico and the number of new enzymes described experimentally, with the proportion of the latter ones approaching 0% [38,39], despite successful approaches of enzyme screen programmes through metagenomic approaches [40].

In addition to genomic screenings, recent technological developments in the complementary fields of transcriptomics have developed tools suitable for further discoveries. Indeed, advances within the field of RNA sequencing [41] have allowed analysis of whole (meta)transcriptomes and made them available for the generation of high-quality protein sequence databases, facilitating enzyme identification. The functional annotation of selected genomes and transcriptomes is relevant to infer associated biological functions and application of proteomics studies in the discovery of bioactive proteins. 

As for proteomics approaches, the most commonly used method for enzyme discovery is bottom-up shotgun proteomics [42], in which proteins are enzymatically digested and the obtained peptides are analysed either by liquid chromatography coupled to electrospray ionization (ESI–LC–MS/MS) or by matrix-assisted laser desorption/ionization followed by time of flight mass spectrometry (MALDI–TOF–MS). A further direct method for enzyme discovery is activity-based proteomics, which takes advantage of enzyme class-specific substrates for the identification of groups of enzymes [43,44].

### Enzyme Engineering

The ultimate value of biotechnology is the production and delivery of molecules or processes of interest for many applications; the emerging requirement for biotechnological products is to be energy saving, thus reducing environmental impacts [45]. Therefore, enzymes from cold-adapted organisms are important because their properties meet the ongoing efforts and needs to decrease energy consumption [46].

The ability of enzymes to catalyze a diverse set of reactions with exquisite specificity makes these proteins essential for reactions useful to humankind. Due their structural and functional complexity, the use of natural enzymes in biotechnology has been limited over the past decades. In fact, natural enzymes generally require modification for industrial use [47], given the improvements in catalytic efficiency, stereo-selectivity, and stability that can be reached through combinatorial engineering strategies [48]. Recent advances in science and technology have led to activity improvements for native and non-native substrates, synthesis of new chemistries and functional characterization of promising novel enzymes. 

In the past, enzyme-based industrial processes were designed around the limitations of natural enzymes discovered in nature; today, enzymes can be engineered to fit the process specifications through enzyme engineering [1]. Enzyme engineering can be pursued by either rational site-directed mutagenesis based on currently available sequence and 3D structures deposited in the RCSB Protein Data Bank, or by directed evolution. Directed evolution is a key technology at the base of the development of new industrial biocatalysts [49,50,51,52] by random amino acid residues changes in the enzyme, largely carried out using PCR-based methods, followed by selection or screening of resulting libraries for variants with improved enzymatic properties. Changes in enzyme properties usually require simultaneous multiple amino-acid substitutions, creating exponentially more variants for testing. However, modern high-throughput screening methods, such as fluorescence-activated cell sorting [53,54,55] allow the screening of tens of millions of variants in a short time. Moreover, the best approach to create multiple mutations is to limit the choices by statistical or bioinformatic methods, such as the statistical correlation approach based on the ProSAR (protein structure-activity relationship) algorithm [56], to identify if a particular substitution is beneficial or not.

Furthermore, the development of synthetic biology methodology, through which large DNA sequences can be synthesized de novo, is increasing the ability for designing and engineering enzymes with new functions. Moreover, the possibility to synthesize whole-gene synthesis can also be used to build high-quality DNA libraries [1]. 

## 3. Cold-Adapted Enzymes and Their Biotechnological Applications

The bioprospecting of microorganisms living in extreme environmental conditions has already led to the discovery of several enzymes endowed with catalytic properties potentially useful for biotechnological applications [57], including some isolated from marine polar regions [23]. Moreover, the systematic investigation of the relationship between the structure and function of these enzymes—in comparison with their mesophilic homologs—has allowed recognising specific structural features associated with cold adaptation, which can be applied to the design of non-natural enzymes endowed with high activity at low/room temperatures. New catalytic activities are expected to arise from the Enzyme Function Initiative (EFI), which was recently established to address the challenge of assigning reliable functions to enzymes discovered in bacterial genome projects [58]. The main aim of the initiative is the computation-based prediction of substrate specificity for functional assignment of unknown enzymes.

### 3.1. Applications of Cold-Adapted Enzymes

The ultimate value of biotechnology is the production and delivery of molecules or processes of interest for many applications; the emerging requirement for biotechnological products is to be energy saving, thus reducing environmental impacts. Therefore, enzymes from cold-adapted organisms are important because their properties meet the ongoing efforts and needs to decrease energy consumption.

The ability of enzymes to catalyze a diverse set of reactions with exquisite specificity makes these proteins essential for biochemical studies, as well as promising catalysts for reactions useful to humankind. Due to their structural and functional complexity, the use of natural enzymes in biotechnology has been limited over the past decades. In fact, natural enzymes generally require modification for industrial use, given the improvements in catalytic efficiency, stereo-selectivity, and stability that can be reached through combinatorial engineering strategies. Recent advances in science and technology have led to activity improvements for native and non-native substrates, synthesis of new chemistries and functional characterization of promising novel enzymes. 

Protein engineering technologies make it possible to develop enzymes that are highly active on non-natural targets, in the presence of organic solvents, and even enzymes for chemical transformations not found in nature. Application of these advanced engineering technologies to the creation of biocatalysts typically starts with an integrated approach with iterative cycles of DNA mutation and selection to create—by directed evolution—enzymes with novel functions for biotechnological approaches [59]. Due to our enhanced understanding of how these enzymes work in extreme environments, we can look forward to increasingly use computational tools, such as Artificial Intelligence, and to design enzymes with new structures and functions [60].

Cold-adapted enzymes have been suggested as biotechnological tools for several reasons:

(1) They are cost-effective, e.g., lower amounts are required, due to higher catalytic efficiency at low temperature;
(2) They can catalyze reactions at temperatures where competitive, undesirable chemical reactions are slowed down. This property is particularly relevant in the food industry, where deterioration and loss of thermolabile nutrients can occur at room temperature;
(3) They catalyze the desired reactions at temperatures where bacterial contamination is reduced. There is a number of advantages in working at lower temperature (around 10–15 °C) than those currently used for large-scale industrial production;
(4) Most cold-adapted enzymes can be inactivated by moderate heat due to their thermolability, avoiding chemical-based inactivation. A striking application of this property has been described for the design of live vaccines. Mesophilic pathogens were engineered for production of thermolabile homologs of essential enzymes, making them temperature-sensitive (TS). The engineered strains are inactivated at mammalian body temperatures, thus losing pathogenicity, but retaining their entire antigenic repertoire. Duplantis and colleagues [61] were able to entirely shift the lifestyle of *Francisella* (*F. novicida*), responsible for tularaemia disease in mice, by substituting its genes encoding essential enzymes with those identified in an Arctic bacterium. The authors applied the same approach to *Salmonella enterica* and the Gram-positive *Mycobacterium* [62]. The TS *S. enterica* strains were shown to be safe in research or diagnostic laboratories but were still capable of stimulating a protective immune response [63]. The TS strain of *M. tuberculosis* could be a safe research or diagnostic strain that is incapable of causing serious disease in humans while being identical to wild-type *M. tuberculosis* except for the TS phenotype [62].

The isolation of cold-adapted enzymes from natural sources is mostly unfeasible and their recombinant expression through standard protocols is hindered by the relatively high growth temperature of the commonly used expression hosts, including *E. coli*. However, several recombinant expression systems have been devized for the production of cold-adapted enzymes [64], mostly aimed at the stabilization and solubilization of heat-sensitive proteins. Development of low-temperature expression system has been attempted in the psychrophilic *Arthrobacter* sp. isolated from a Greenland glacier [65]. An alternative strategy is to use the engineered Arctic Express Cells which co-express cold-adapted homologues of the *E. coli* GroELS chaperonines, Cpn60 and Cpn10, from *O. antarctica* [66]. This system allows protein processing at very low temperatures (4–12 °C), potentially increasing the yield of active, soluble recombinant protein.

### 3.2. Structural Features of Cold-Adapted Enzymes

A basic requirement of evolutionary cold adaptation is that the enzyme should afford metabolic rates comparable to those of mesophilic organisms at its working temperature. Indeed, the temperature-dependence of the catalyzed reaction constants is described by the Arrhenius Equation (1)

k = Ae^−E/(RT)^ (1)

where k is the reaction rate, E is the activation energy of the reaction, R is the gas constant, T is temperature and A is a collision frequency factor. It shows that a decrease of 10 °C brings about a decrease of 2–3 fold in the reaction rate. Therefore, a mesophilic enzyme with an optimal working temperature around 37 °C is expected to work 16–80 times slower when brought to 0 °C [67]. Increases in enzyme expression in psychrophilic microorganisms to compensate for the slower reactivity have been observed only in a small number of cases. Rather, the most common evolutionary adaptation consists in the evolution of enzyme homologs endowed with increased catalytic efficiency, the k_cat_/K_M_ ratio, either by an increase of k_cat_—particularly for enzymes working at saturating concentrations of substrate—or by a decrease in K_M_—particularly for enzymes working at sub-saturating concentrations of substrate. k_cat_ is almost invariably increased in cold-adapted enzymes, up to 10-fold. The trade-off between k_cat_ and K_M_ in cold-adapted enzymes has been comprehensively reviewed [68].

From a structural point of view, it is widely accepted that these kinetic parameters are associated with the increased flexibility of these enzymes [69,70]. This dynamics adaptation allows for the conformational rearrangements required for catalysis at low temperatures, making it possible for cold-adapted enzymes to maintain the same conformational space accessible to their mesophilic and thermophilic homologs at higher temperatures. Indeed, for the latter enzymes, the kinetic energy associated with low temperatures would be insufficient to overcome the kinetic barriers required for the conformational rearrangements associated with catalysis. The turnover number, or k_cat_, is a good index of enzyme flexibility, as it reflects the rate of transition between all conformational states involved in the catalytic cycle. 

Multiple structural features are usually associated with increased flexibility, involving both the active site and the peripheral regions. Commonly observed structural features are: Decreased core hydrophobicity, fewer prolyl residues in loops, increased hydrophobicity of the surface, more glycyl residues, lower arginyl/lysyl ratio, weaker interactions between subunits and domains, longer loops, decreased secondary-structure content, more prolyl residues in α-helices, fewer and weaker metal-binding sites, fewer disulfide bridges, fewer electrostatic interactions, reduced oligomerization and increase in conformational entropy of the unfolded state [71,72]. Many of these structural adaptations are summarized in Table 1. The crystal structures of 11 proteins isolated from the Antarctic marine oil-degrading bacterium *Oleispira antarctica* (α/β hydrolase, phosphodiesterase, transaldolase, isochorismatase, amidohydrolase, fumarylacetoacetate isomerase/hydrolase, 2-keto-3-deoxy-6-phosphogluconate aldolase, phoshonoacetaldehyde hydrolase, inorganic pyrophosphatase, and protein with unknown function) revealed that the most dominant structural feature is an increase in surface hydrophobicity and negative charge, with higher Glu+Asp/Arg+Lys ratio compared to their mesophilic counterparts [73]. 

In other instances, however, a small number of specific residues was recognized as solely responsible for the cold adaptation of enzymes. In the β-galactosidase from the polyextremophilic Antarctic archaeon *Halorubrum lacusprofundi*, the mutation of only six amino-acid residues in comparison to its mesophilic homologs resulted in altered temperature activity profiles, suggesting that a small number of mutations can indeed account for cold adaptation [71]. In other proteins, a limited number of regions were associated to cold adaptation: the characterization of chimeric derivatives of monomeric isocitrate dehydrogenases of *Colwellia maris* and *Pseudomonas psychrophila*—cold adapted and mesophilic, respectively—led to the identification of two regions responsible for their different thermal properties [78]. The relative contribution of local and global protein flexibility in determining cold adaptation was assessed by performing molecular dynamics simulations on cytosolic malate dehydrogenases orthologous from marine mollusks adapted to different temperatures [79]. A convergent evolution trend was observed, with a significant negative correlation between the adaptation temperature of the organism and overall protein flexibility, with regions involved in ligand binding and catalysis showing the largest fluctuation differences [79].

Generally, the higher flexibility at low temperatures brings about low stability at higher temperatures. However, thermolability, although common [70], is not universal in cold-adapted enzymes. Three superoxide dismutases from the Antarctic psychrophilic ciliate *Euplotes focardii* were recently showed to exhibit a melting temperature of around 50–70 °C, suggesting a combination of cold adaptation and relative tolerance to high temperatures [80]. GroEL and thioredoxin from the Antarctic bacterium *Pseudoalteromonas haloplanktis* TAC125 were shown to be only marginally less stable than their *Escherichia coli* homologs [81].

It should also be highlighted that cold adaptation does not only consist in altered kinetic parameters of individual enzymes. For instance, sequencing and functional analysis of the genome the Antarctic marine oil-degrading bacterium *O. antarctica* revealed an array of alkane monooxygenases, osmoprotectants, siderophores and micronutrient-scavenging pathways [73] associated to cold adaptation.

### 3.3. Examples of Biotechnological Applications of Polar Enzymes 

According to the type of catalyzed reaction, enzymes are classified into six main classes, i.e., oxidoreductases, transferases, hydrolases, lyases, isomerases and ligases, as reported in Table 2. The proposed biotechnological applications of cold-adapted enzymes belonging to several of these classes have been extensively reviewed [23,25,82,83,84,85].

Currently, approximately 65% of more than 3000 enzymes with an industrial application are hydrolases, used in the detergent, textile, paper, and food industries [167,191]. Hydrolases, classified as Class 3 (EC 3) by the Nomenclature Committee of the International Union of Biochemistry and Molecular Biology (NC-IUBMB), catalyze the hydrolysis of chemical bonds. Herein, we focus on some recent examples of cold-adapted enzymes, particularly hydrolases, isolated from marine polar microorganisms. More examples of microbial enzymes isolated in Arctic or Antarctic marine polar regions are reported in Table 2. To our knowledge, this is the first table including only cold-active enzymes isolated from marine polar microorganisms. Indeed, several recent reviews do not distinguish between polar and non-polar enzymes or between marine and non-marine origin. The latter distinction is crucial to guide bioprospecting in search for novel activities.

#### 3.3.1. Glycoside Hydrolases

**Scheme 1:**
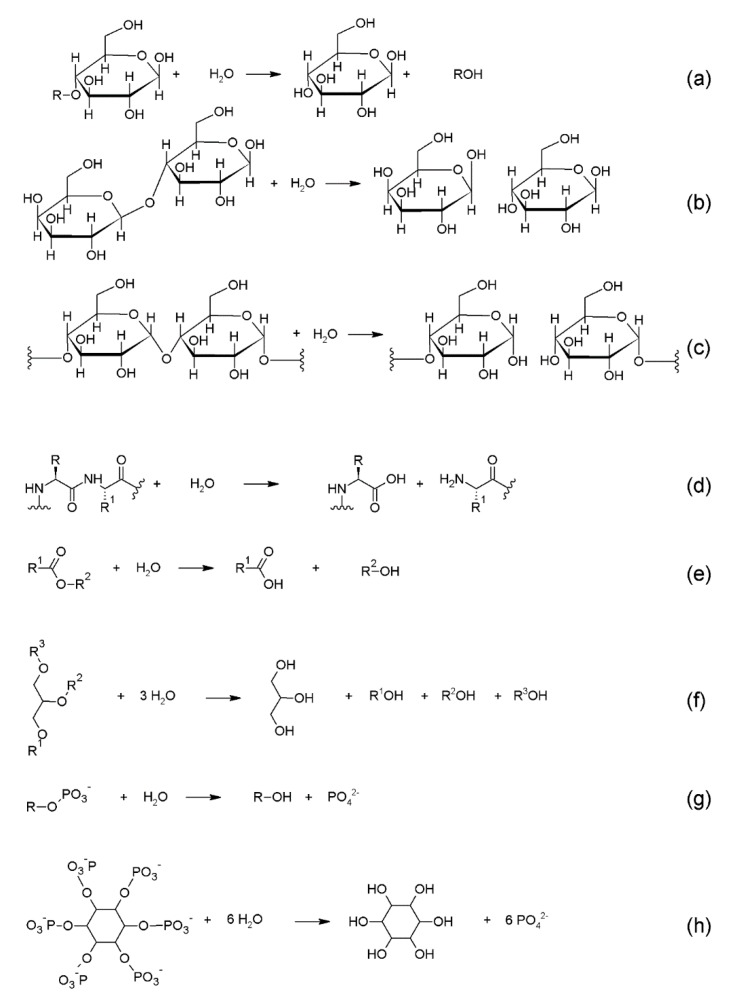
Principal enzymatic activities of cold-adapted enzymes investigated for biotechnological applications. (**a**) Generic reaction catalyzed by glycoside hydrolases, with a glucose derivative as substrate. (**b**) Reaction catalyzed by lactases, with conversion of lactose to galactose and glucose. (**c**) Reaction catalyzed by amylases on glucose polymers, with glucose or maltooligosaccharides as final products. (**d**) Generic reaction catalyzed by proteases, which catalyze the hydrolysis of peptide bonds of proteins or peptides. (**e**) Generic reaction catalyzed by esterases, which hydrolyse ester bonds. (**f**) Reaction catalyzed by lipases, with the hydrolysis of fatty acids from acyl glycerol. In this case, the full hydrolysis of a triglyceride to glycerol is represented. (**g**) Generic reaction catalyzed by phosphatases with the hydrolysis of phosphate monoesters. (**h**) Reaction catalyzed by phytases. In this case, the full hydrolysis to phosphate ions and inositol is represented.

Glycoside hydrolases (GH; EC 3.2.1.), or glycosidases, hydrolyse the glycosidic bond between two or more carbohydrates, or between a carbohydrate and a non-carbohydrate moiety (Scheme 1a), overall processing a wide variety of complex substrates [192]. They are extremely common in nature, with more than 100 GH families currently recognized and grouped in the Carbohydrate-Active Enzymes database (CAZy), which provides a continuously updated list of families [193]. Their function ranges from the degradation of complex carbohydrates for their metabolic use (cellulases, amylases), to the defense against pathogens (lysozyme, chitosanase), to the synthesis of the oligosaccharide groups of glycoproteins. Their biotechnological applications are manifold, especially in the food processing industry, where enzyme activity at low temperatures is desirable to slow down spoilage and loss of nutrients. For this reason, cold-adapted hydrolases are usually in need [194]. 

Lactases or β-galactosidases (EC 3.2.1.23) (Scheme 1b) offer a clear example in this respect: with more than 70% of the world population suffering from lactose intolerance, the enzymatic removal of lactose from milk and dairy products using β-galactosidases is now common. The enzyme catalysing the hydrolysis of lactose into its constituent monosaccharides, glucose, and galactose, has drawn the interest of many research groups and enterprizes due to nutritional (lactose intolerance) and technological (crystallization), challenges associated with lactose [195]. The isolation of several cold-adapted β-galactosidases has been reported. Among them, some are from Antarctic organisms, e.g., *H. lacusprofundi*, isolated from the hypersaline Deep Lake [71,196], *Arthrobacter* species from soil samples [197,198], and marine *Pseudoalteromonas* species [86,87,88,199]. Other enzymes were isolated from marine organisms from the Arctic region, including those from *Enterobacter ludwigii* [91] and *Alkalilactibacillus ikkense* [92]. A cold-active β-galactosidase from the Antarctic marine bacterium *P. haloplanktis* TAE 79 was applied to lactose hydrolysis at low temperatures during milk storage [88] and was proposed for the production of tagatose, a natural monosaccharide with low caloric value and glycemic index [200]. A cold-active lactase isolated from the Antarctic marine *P. haloplanktis* LMG P-19143 was patented [89] and is also produced in large quantities by Nutrilab NV (Bekkevoort, Belgium). Yeasts are important sources for β-galactosidase production. *Guehomyces pullulans* 17-1, isolated from the Antarctic sea sediment, synthesize both extracellular and cell-bound β-galactosidases [90]. A 1,3-α-3,6-anhydro-L-galactosidase—an enzyme involved in the final steps of the agarolytic pathway and used in the industrial processing of agar—was identified in the agarolytic marine bacterium *Gayadomonas joobiniege* G7 and was shown to be active between 7.0 and 15 C [201].

Amylases (Scheme 1c) are also interesting enzymes for industrial processes, in detergent formulations and in food industry for beer and wine fermentation and for the preparation of bread and fruit juice. They hydrolyse starch to maltose, maltotriose, glucose monomers and limit dextrins. They can be classified in exoamylases (β-amylase EC 3.2.1.2; glucoamylase, EC 3.2.1.3; α-glucosidase, EC 3.2.1.20) endoamylases (α-amylase, EC 3.2.1.1) and debranching (pullulanases, EC 3.2.1.41; isoamylases, EC 3.2.1.68; dextrinases, EC 3.2.1.142) according to the specificity of the hydrolysis reaction they catalyze [194]. In the detergent industry, cold-active amylases not isolated from polar regions are already available on the market [167], i.e., Stainzyme^®^ (active at temperatures between 30 and 70 °C) and Stainzyme^®^ Plus (active below 20 °C) released by Novozymes and Preferenz™ S100 (active at 16 °C) released by DuPont Industrial Biosciences. In the textile industry, Genencor-DuPont developed Optisize^®^ COOL, using a cold-active amylase for the desizing of woven fabrics at low temperatures. Other cold-active, detergent-stable α-amylases, not isolated from polar regions, were identified from *Bacillus cereus* GA6 [202] and from the marine bacterium *Zunongwangia profunda* [203]. The latter was isolated from surface seawater in the costal area of Fujian, China, but shows a cold-adapted and salt-tolerant α-amylase activity. An α-amylase produced by the psychrophilic yeast *Glaciozyma antarctica* PI12 was recently characterized [94].

Cold-adapted enzymes from marine polar regions could successfully be employed in these applications, and some of them are already under investigation or in use. A cold-adapted α-amylases from the Antarctic sea-ice bacterium *Pseudoalteromonas* sp. M175 exhibited resistance towards all the tested commercial detergents and was shown to improve their stain removal efficiency [93]. Heat-labile α-amylases displaying high activity at low temperature isolated from bacteria of Antarctic seawater were structurally studied [96,97].

Pullulanases (EC 3.2.1.41), debranching enzymes that hydrolyse α-1,6-and α-1,4-linkages in pullulan, starch, amylopectin and various related oligosaccharides are interesting enzymes in starch processing. There are different pullulanase groups according to their substrate specificities and reaction products. Pul13A is a type-I pullulanase isolated from Arctic seawater strain *Shewanella arctica* in Spitsbergen, Norway. It is able to hydrolase α-1,6-glycosidic bonds in pullulan to produce maltotriose at low temperature, displaying low thermostability at elevated temperatures. It represents a potential candidate for industrial applications such as starch degradation for ethanol-based biofuel production [143].

Although poorly studied, cold-active xylanases (EC 3.2.1.8) are also interesting in the food industry for bread making, as they convert the insoluble hemicellulose of dough into soluble sugars, thus yielding soft and elastic bread. Three cold-adapted xylanases from psychrophilic bacteria were shown to improve dough properties and bread volume with respect to mesophilic orthologues [204]. The xylanase pXyl from *P. haloplanktis,* isolated from soil samples [205], was very efficient in improving the dough properties and bread volume due to its high activity at low temperature [205]. The psychrophilic enzyme is now sold by Puratos (Grand-Bigard, Belgium). Antarctic marine fungi from marine sponges, which are the dominant macroinvertebrates in many benthic communities, are considered new and promising sources of cold-active xylanases. *Cladosporium* sp. isolated from marine sponges collected in King George Island, Antarctica, showed high xylanase activity at low temperature and very low thermal stability [99]. Also *Flavobacterium frigidarium* sp. nov., an aerobic psychrophilic and halotolerant bacterium isolated from marine sediment of shallow waters surrounding Adelaide Island in Antarctica, exhibited xylanolytic activity [100].

Cellulases, used in detergents for color and brightness care, have been classified according to their activity into endo- (EC 3.2.1.4) and exo-cellulases with exo-β-1,4-glucan cellobiohydrolase (EC 3.2.1.91) and β-glucosidase (EC 3.2.1.21). *P. haloplanktis* is able to convert the cellulose into an immediate nutritive compound for plants by hydrolysis. *P. haloplanktis* secretes a multi-modular endocellulase composed N-terminal catalytic module, a linker region and the cellulose-binding module. Structural adaptations to cellulose hydrolysis at low temperatures have been identified in the catalytic module and unusually long linker region by using both X-ray diffraction and small angle X-ray scattering methods [145]. 

*Leucosporidium antarcticum* strain 171, widespread in the cold marine waters below the Antarctic ice, was isolated from a seawater sample collected at a depth of 100 m in Admiralty Bay, King George Island. The yeast was found to produce cold-adapted invertases (β-D-fructofuranoside fructohydrolases, EC 3.2.1.26) and glucosidases (α-D-glucoside glucohydrolases, EC 3.2.1.20). Their synthesis may facilitate assimilation of β-fructofuranosides and α-glucopyranosides in cold environments where nutrient availability is fluctuating [144].

Chitin, one of the most abundant organic compounds in nature, is a structural polysaccharide composed of N-acetylglucosamine (GlcNAc) residues. Chitobiases (EC 3.2.1.29) found in bacteria, fungi, and eukaryotes, hydrolyse chitobiose to GlcNAc, which is further converted to glucosamine by a deacetylase. A cell-bound chitobiase was isolated from the marine psychrophile *Arthrobacter* sp. TAD20 collected along the Antarctic ice. The cold-adapted chitobiase overexpressed in *E. coli* displayed four functionally independent domains: (i) The catalytic domain, (ii) the galactose-binding domain, and (iii) the immunoglobulin-like domain followed by (iv) the cell-wall anchorage signal. The enzyme exhibited features that are typical of cold-adapted enzymes, with unusually low-K_m_ and high-k_cat_ values, with improved flexibility around the active site for efficient activity at low temperatures [146].

#### 3.3.2. Proteases

Proteases (EC 3.4) catalyze the hydrolysis of peptide bonds (Scheme 1d). This catalytic activity has evolved multiple times, yielding enzymes with different catalytic mechanisms [206]. These enzymes are classified as exo-peptidases (EC 3.4.11-19), when they cleave the terminal amino acid, and as endo-peptidases (EC 3.4.21-99), when the peptide bonds they hydrolysed is internal. Proteases have several industrial applications, particularly in the food industry and as components of laundry detergents [207,208]. Both thermophilic [209] and cold-adapted homologs [210] are of biotechnological interest, depending on the intended working temperature of the process. To obtain cold-active proteases, two approaches have been pursed: mutagenesis of mesophilic enzymes or identification and expression of naturally occurring psychrophilic proteases. The former approach was applied to subtilisins from *Bacillus* species, mutated to increase their activity at low temperature. Particularly, the subtilisin savinase from *Bacillus* was mutated to produce a thermostable variant with increased activity at low temperature [211]. The latter approach has also been investigated. 

Commercially exploited cold-adapted proteases include a protease from *Bacillus amyloliquefaciens—*a non-polar soil bacterium—commercialized as Purafect Prime L^TM^, which is active at 20 °C, and the protease Properase^TM^ from *B. alcalophilus*, which combines activity at high pH and low temperature and Excellase^TM^. They are commercialized by Genencor (Genencor International Inc., Palo Alto, CA, USA). A cold-adapted extracellular aspartic protease was isolated from the yeast *Sporobolomyces roseus* isolated from an underground water sample drawn from the disused silver and lead mine “Luiza” (Zabrze, Poland) and was proposed as biocatalyst in the food industry, particularly in cheese and soy-sauce production, meat tenderization, and as bread additive [212]. It was also proposed in the production of antioxidant peptides from dairy and animal proteins, as hydrolysis of beef casein by this enzyme yielded peptides endowed with antioxidant activity [212].

Bioprospecting of cold-adapted bacteria from the polar regions could further increase the variety of cold-active proteases for industrial use. Several recent examples of such enzymes have been reported. Particularly, Antarctic fungi capable of producing proteases have been recently reviewed [85]. Two cold-adapted subtilisins from Antarctic marine *Bacillus* TA39 [102] and TA41 [103] showed high activity at low temperatures but also limited stability. A mutant with unaltered activity at low temperatures but higher stability was obtained through directed evolution [101]. A subtilisin-like cold-adapted serine peptidase was isolated from the Antarctic bacterium *Lysobacter* sp. A03, and is both active at low temperatures and resistant to higher temperatures [113]. 

A protease from the Antarctic bacterium *Janthinobacterium lividum* obtained from the Polar BioCenter (Korea Polar Research Institute) [213], recombinantly expressed and purified, was reported. Among the fungal proteases identified from polar marine samples, the extracellular subtilase lap2, from the Antarctic yeast *Leucosporidium antarcticum* 171, exhibited a low optimal temperature (25 °C) poor thermal stability, and high catalytic efficiency in the temperature range 0–25 °C [109]. 

The extracellular protease released by *Rhodotorula mucilaginosa* L7 strain was isolated from an Antarctic marine alga and was shown to be stable in the presence of high concentrations of NaCl [106]. A new cold-adapted protease with a potential biotechnological application, largely represented amongst Antarctic bacterial genus (*Pseudoalteromonas* sp., *Marinobacter* sp., *Psychrobacter* sp., *Polaribacter* sp.), exhibits a higher catalytic efficiency at lower temperatures compared to its mesophilic counterpart [107]. The extracellular cold-active protease from the marine psychrophilic bacterium *Pseudoalteromonas* sp. NJ276, for its catalytic activity and broad substrate specificities, has a potential application in low-temperature food processing, in particular in the preservation of milk at low temperature [108]. A secreted a cold-active serine protease identified in *Colwellia* sp. NJ341, isolated from Antarctic sea-ice, showing a 30% of activity at 0 °C and a better thermostability than other cold-active proteases, represents a good candidate for industrial applications, particularly in processes where there may be a risk of microbial contamination or a temperature instability of reactants or products [104].

An aminopeptidase produced by the marine psychrophile *Colwellia psychrerythraea* strain 34H was structurally investigated, displaying structural features (fewer proline residues, fewer ion pairs, and lower hydrophobic residue content) related to the cold adaptation and able to increase the flexibility for activity in the cold [111].

Besides their industrial applications, proteases have also long been used for therapeutic applications, particularly in the treatment of cardiovascular diseases, sepsis, digestive disorders, inflammation, cystic fibrosis, retinal disorders, and psoriasis [214]. The proteases so far approved by the US FDA derive from mesophilic organisms. However, the cold-adapted ones are endowed with a higher catalytic efficiency [215] and might, therefore, be of great interest. Indeed, they evolved to be efficient at low temperatures and, if thermostable enough, they would further increase their reaction rates at 37 °C, the intended temperatures for therapeutic applications. To overcome their thermal instability, mutational studies have been conducted [83].

As for general applications in molecular biology, proteinase, an endopeptidase from an Arctic marine microbial source, was developed by ArcticZymes [118] for the digestion of chromatin, thus releasing naked DNA. As it is thermolable, it can be inactivated at temperatures compatible with RNA integrity and DNA as double strands. 

#### 3.3.3. Lipases and Esterases

Esterases (EC 3.1.1) hydrolyse ester bonds (Scheme 1e). Lipases in particular (EC 3.1.1.3) are glycerol-ester hydrolases that catalyze the hydrolysis of triglycerides to fatty acids and glycerol (Scheme 1f). They are widely used in organic synthesis and as laundry detergents [216]. Although thermophilic homologs might appear more promising for industrial applications for their higher stability under harsh conditions, cold-adapted homologs are also actively investigated for their high activity at low temperatures [217]. Moreover, lipases are promising tools for the preparation of chiral molecules in the pharmaceutical industry, where low working temperatures are desirable to reduce the rate of non-catalytic side reactions. Particularly, the predictable enantiopreference of lipases allows the determination of the absolute configuration of secondary alcohols using the lipase-catalyzed kinetic resolution and the use of cold-active lipases in organic solvents is excellent for the preparation of single-isomer chiral drugs, and the enantioselective or regioselective preparation of alcohol and amine intermediates in the synthesis of pharmaceuticals [218]. 

There are already examples of lipases identified in organisms isolated from the polar regions which are currently on the market. Lipozyme^®^ CALB is a non-specific lipase from *Candida antarctica* which was successfully used in the resolution of racemic alcohols, amines, and acids, and in the preparation of optically active compounds from meso substrates, as well as for the regio-selective catalysis in the selective acylation of different carbohydrates [219]. Lipases from *C. antarctica* catalyze hydrolysis on several substrates and are thermostable, a surprising property considering their origin. CALB is currently commercialized by Novozyme (Bagsværd, Denmark). The lipase from *C. antarctica* is also available in an immobilized form and commercialized as Novozym 435 (Novozyme, Bagsværd, Denmark). 

More cold-adapted lipases were recently discovered in Arctic and Antarctic microorganisms. A lipase from the psychrotolerant yeast *Rhodotorula* sp. Y-23 isolated in the Nella Lake, East Antarctica, exhibited the highest V_max_ at 15 °C and high compatibility with commercial detergents, which brought about an increase in activity, making it a potential candidate in detergent formulation active at low temperatures [220]. The Gram-positive psychrotrophic Arctic bacterium *Arthrobacter gangotriensis*, obtained from an soil sample, was recently shown to exhibit lipolytic activity associated with a lipase that can work in a wide pH range and that exhibits good stability [221]. The bacterium *Bacillus pumilus* ArcL5 with lipolytic activity was isolated from the Chukchi Sea within the Arctic Ocean; the lipase BpL5, was recombinantly expressed in *E. coli* and was shown to retain 85% of its activity at 5 °C. Two mutants active against tricaprylin were also characterized [119].

Other lipases were identified in Antarctic soils [222]. The lipase LipG7 from the Antarctic filamentous fungus *Geomyces* sp. P7 was unusually thermostable and was proposed as an enantioselective biocatalyst [223].

Other cold-active lipases were identified in the Antarctic marine *P. haloplanktis* TAC125 [120], in the Arctic marine bacterium *C. psychrerythraea* 34H [121], *Psychrobacter* sp. of Antarctic marine origin [123,124,126] and Antarctic deep-sea water strain *Moritella* sp.2-5-10-1 [127,128].

Esterases (EC 3.1.1.1), hydrolases that catalyze the cleavage of simple esters such as triglycerides with short chains of fatty acids (less than eight carbon atoms) (Scheme 1e) [194], are interesting enzymes in organic synthesis and cheese ripening processes [137]. Cold-adapted esterases were identified in Arctic marine bacteria *Pseudoalteromonas arctica*, [131] and *Thalassospira* sp. [132] and the Antarctic marine bacteria *O. antarctica* [133] and *P. haloplanktis* TAC125 [134,135]. Moreover, a cold-adapted esterase was identified from *Pseudoalteromonas* sp. strain 643A, isolated from the alimentary tract of Antarctic krill *Euphasia superba* Dana. It displayed 20–50% of maximum activity at 0–20 °C, and the optimal temperature was close to 35 °C. The optimal pH for enzyme activity was around 8.0; however, it was stable between pH 9 and 11.5 [136].

#### 3.3.4. Phosphatases

Alkaline phosphatases (EC 3.1.3.1), which catalyze the hydrolysis of phosphate monoesters (Scheme 1g), have an important application in molecular biology for the dephosphorylation of DNA linearized at the 5′ end to avoid its re-circularization during cloning processes. A recombinant alkaline phosphatase isolated from the Antarctic bacterial strain TAB5 [147,148] was developed by New England Biolabs [149]. The crystal structure of a cold-active alkaline phosphatase from a psychrophile, *Shewanella* sp. isolated from the intestine of an Antarctic shellfish, revealed local flexibility, responsible for the high catalytic efficiency at low temperatures [150].

Phytic acid is the phosphate ester of inositol. The phosphate groups in this form are not available for absorption by animals, with the partial exception of ruminants, where the hydrolysis is carried out by the rumen microbiota. Phytases (EC 3.1.3.) have been used for decades to enrich animal food of absorbable phosphate groups, both for non-ruminant livestock and fish [224] (Scheme 1h). Thermophilic phytases have been suggested for industrial use [225], but cold-adapted homologs were also proposed, especially in aquaculture, where low temperatures might be limiting for the activity of the mesophilic orthologues. In this view, the purification and characterization of novel cold-adapted phytases from the Antarctic marine *R. mucilaginosa* strain JMUY14 [130] and *Pseudomonas sp.* strain JPK1 isolated from soil were reported [226].

#### 3.3.5. Other Hydrolases

A cold-adapted uracil-DNA glycosylase (EC 3.2.2.27 and EC 3.2.2.28) of Antarctic marine source was released by New England Biolabs as thermolabile uracil-DNA glycosylase [154]. This hydrolase, isolated from a psychrophilic marine bacterium, catalyzes the release of free uracil from uracil-containing single-stranded or double-stranded DNA; it is sensitive to heat and can be inactivated at temperatures above 50 °C. 

Cryonase, a cold-active nuclease, isolated from an Antarctic marine psychrophile, *Shewanella* sp. strain Ac10 and patented by Awazu and colleagues [227] was developed by Takara-Clontech [151]. It is a recombinant endonuclease that can digest all types of DNA and RNA (single-stranded, double-stranded, linear or circularized) at low temperatures, frequently used during DNA digestion of samples in the presence of heat-labile proteins. A 3′–5′ exonuclease specific for single-stranded DNA and derived from an Arctic marine bacterium was released by ArcticZymes [152]. 

Antimicrobial enzymes endowed with bactericidal or bacteriostatic properties are an emerging strategy to combat pathogens, particularly by degrading their DNA, polysaccharides, and proteins, or by interfering with biofilm formation, or by catalysing reactions which result in the production of antimicrobial compounds [228]. For example, N-acyl homoserine lactones are signaling molecules involved in bacterial quorum sensing and their hydrolysis can reduce growth and biofilm formation of bacterial pathogens. A cold-adapted N-acylhomoserine lactonase (EC 3.1.1.81) was recently identified in Antarctic *Planococcus* sp. isolated from a soil sample collected on Lagoon Island (Antarctica) and was shown to attenuate the pathogenicity of *Pectobacterium carotovorum*, a plant pathogen that causes soft-rot disease [229]. As the enzyme was shown to be thermolabile at the human body temperature, it was suggested as a safe antimicrobial agent in the treatment of crops, as it would be inactivated upon ingestion. 

#### 3.3.6. Other Enzymes

Besides hydrolases, other enzymes have been isolated in marine microorganisms from the polar regions and, for some of them, a biotechnological application has been proposed. 

Recently, few enzymes showing signatures of cold adaptation in their activity and structure have been isolated and fully characterized from the Arctic marine bacterium *C. psychrerythraea* strain 34H and also included a phenylalanine hydroxylase (PAH), an oxidoreductase, with high-catalytic efficiency at 10 °C, high thermostability and low affinity for substrate, probably due to enhanced flexibility of the active site [76]. PAH (EC 1.14.16.1) catalyzes the conversion of L-Phe to L-Tyr by para-hydroxylation of the aromatic side-chain and recently there has been an increased number of studies on the structure and function of PAH from bacteria and lower eukaryote organisms. Almost all characterized bacterial PAHs are monomeric and display a fold similar to the catalytic domain of mammalian PAHs.

Many oxidoreductases that catalyze redox reactions of industrial interest have been described in cold-adapted bacteria. The crystal structure of a Fe-superoxide dismutase, Fe-SOD (EC 1.15.1.1) isolated from the marine *P. haloplanktis*, revelead that cold-adapted enzyme displays high catalysis at low temperature increasing the flexibility of its active site without modifying the overall structure [161]. SODs have been used in the pharmaceutical and cosmetic industries, food, agricultural, and chemical industries [169]. Antioxidant defense is an important component of evolutionary adaptations in the cold to face increased levels of reactive oxygen species (ROS). The cold waters of the polar regions promote the formation of ROS and would be expected to lead to enhanced ROS damage of DNA and membrane lipid peroxidation in polar species [72]. *P. haloplanktis* TAC125 copes with increased O_2_ solubility by deleting entire metabolic pathways that generate ROS as side products [230]. In contrast, the Arctic bacterium *C. psychrerythraea* has developed an enhanced antioxidant capacity, owing to the presence of three copies of catalase genes, as well as two superoxide-dismutase genes, one of which codes for a nickel-containing superoxide-dismutase, never reported before in proteobacteria [231].

A recombinant form of glutathione reductase (EC 1.8.1.7) from the Antarctic *C. psychrerythraea*, was recently characterized [168]. Since the cold-adapted enzyme displays activity also at moderate temperatures when overexpressed in *E. coli,* it may have some potential for industrial applications to protect cells and tissues from oxidative stress. Malato dehydrogenase (MDH) (EC 1.1.1.37) was purified from the Antarctic marine bacterium *Flavobacterium frigidimaris* KUC-1 and, among cold-adapted MDHs, was shown to be the most thermolabile and cold-active [158]. Thioredoxin and thioredoxin reductase (EC 1.8.1.9) from *P. haloplanktis* TAC125 were obtained as recombinant proteins. Both proteins exhibit activity at 10 °C [170]. The recombinat cold-adapted peroxiredoxin (EC 1.11.1.15) was biochemically characterized from the Antarctic marine psychrophilic bacterium *Psychrobacter* sp. ANT206, which had an optimum growth temperature of 10–12 °C. These enzymes may be relevant for many applications in food and medicine, mostly for their ability to protect super-coiled DNA from oxidative stress [172].

A cold-adapted leucine dehydrogenase (EC 1.4.1.9), a NAD^+^-dependent oxidoreductase, with unique substrate specificity was cloned from the Antarctic marine psychrotrophic bacterium *Pseudoalteromonas* sp. ANT178 and was shown to retain 40% of its maximal activity at 0 °C. Being the key enzyme in the enzymatic conversion of L-leucine and other branched chain L-amino acids in the corresponding α-keto acid, it was suggested as biocatalyst in the pharmaceutical industry [157].

Aminotransferases (EC 2.6.1.), belonging to the class of transferases, have also been isolated from marine polar microorganisms, although no industrial application has been suggested so far. The aspartate aminotransferase, a ubiquitous transaminase enzyme that catalyzes the conversion of aspartate and α-ketoglutarate to oxaloacetate and glutamate, was isolated from the marine psychrophilic bacterium *P. haloplanktis* TAC125 and characterized from a structural and functional point of view [173]. It was shown to be thermolable, being inactivated at 50 °C. Its optimal working temperature is 10 °C lower than its *E. coli* homolog. 

Photolyases (EC 4.1.99.3) were identified in the genera *Pseudomonas*, *Hymenobacter* and *Sphingomonas*, isolated in fresh waters in Antarctica (King George Island, Fildes Peninsula). Present in all living forms, except placental mammals and some marsupials, they are resistant to UV-radiations, being able to reverse DNA lesions (cyclobutane pyrimidine dimers and pyrimidine photoproducts [232] and, as UV radiations are known to be linked to skin cancer, they may have some potential in the cosmetic and pharmaceutical industries.

Cold-active γ-carbonic anhydrases (EC 4.2.1.1), able to catalyze the physiologic reaction of CO_2_ hydration to bicarbonate and protons, were isolated from the psychrophilic marine bacteria *C. psychrerythraea* [178] and *P. haloplanktis* [179] cloned and characterized. 

A cold-active DNA ligase from the psychrophile *P. haloplanktis* TAE72, isolated from Antarctic seawater at the Dumont d’Urville Antarctic Station, displays activity at temperatures as low as 4 °C [177]. DNA ligases (EC 6.5.1.) are enzymes involved in DNA replication, DNA recombination and DNA repair. They are commonly used in molecular biology to catalyze the formation of a phosphodiester bond between adjacent 5′-phosphoryl and 3′-hydroxyl groups in double stranded DNA. Currently, DNA ligases on the market, such as the recombinant versions of bacteriophage-derived DNA ligases, T4 and T7 ligases, and *E. coli* DNA ligases, are enzymes active at temperatures above 15 °C, where residual nucleases may interfere with the ligation process [167]. The possibility to use psychrophilic DNA ligases active at low temperature may guarantee high specific activity at low temperatures, thus avoiding unwanted reactions, carrying out the reaction in shorter times with respect to mesophilic ligases. 

The crystal structure of the sedoheptulose 7-phosphate isomerase from the marine psychrophilic organism *C. psychrerythraea* 34H was determined. The Arctic bacterium produces extracellular polysaccharide substances to cope with cold and the isomerase is essential for producing D-glycero-D-mannoheptose 7-phosphate, a key mediator in the lipopolysaccharide biosynthetic pathway [184]. Other polar enzymes belonging to the class of isomerase are triose phosphate isomerases (EC 5.3.1.1), enzymes involved in the glycolytic pathway, identified in the Antarctic marine bacteria *Pseudomonas* sp. π9 [185], isolated from sea-ice and *Moraxella* sp. TA137 [186], isolated from the intestine of fish caught in Terre d’Adelie in Antarctica.

## 4. Perspectives and Conclusions

Marine microorganisms, whose immense genetic and biochemical diversity is only beginning to be appreciated, may become a rich source of novel enzyme activities in the near future. The marine bioprospecting of polar regions has begun only relatively recently but has already yielded success stories. Taking into account the evidence that the total number of species, and thus most likely biochemical diversity in the oceans, is higher than on land, there is a good reason to believe that a larger number of marine natural products from low-temperature environments may reach different sectors of biotechnology in the near future. For those reasons, a large number of research and development programmes in oceans are in progress worldwide. Although the genomic, functional and physiological knowledge of individual organisms is necessary to understand how enzymes and products work, more efficient strategies are needed to overcome the limits of the cultivation of microbes, especially those living in extreme environments such as the polar ones. The specific properties of polar marine microorganisms that make them unique and biotechnologically interesting are also responsible for their resistance to handling and cultivation. The most significant challenges are (i) the current lack of ability to produce extreme compounds (including enzymes) on large scale; (ii) the need to generate stronger synergies among marine scientists and industries at earlier levels to compensate the chronic underfunding of basic research and before large-scale production; (iii) the need to develop or improve technology transfer pathways for data and to secure access to fair and equitable benefit sharing of marine genetic resources. Metagenomics, genome engineering and systems biology will be fundamental to efficiently produce larger quantities of known and novel bioresources.

In the race to find new products for biotechnology, although there are good reasons to be optimistic, diverse strategies need to be adequately supported and funded in the academic context. The future perspectives require active collaboration between academia and industry at the beginning to support the research thus reducing the time lapse from the discovery to the industrial applications.

## Figures and Tables

**Table 1:** Structural adaptations in cold-adapted enzymes and their effects on protein structure.

Molecular Adaptation	Effect	Reference
Decreased number of hydrogen bonds and salt bridges	Increased flexibility	[69,72]
Reduced proline and arginine content	Increased molecular entropy	[23,74]
Increased surface charged residues	Increased conformational flexibility	[23]
Reduced frequency of surface, inter-domain and inter-subunit ionic linkages and ion-network	Increased conformational flexibility and reduced enthalphic contribution to stability	[75]
Reduced core hydrophobicity/increased surface hydrophobicity	Reduced hydrophobic effect/ entropic destabilization	[70]
Increased accessibility of active site	Increased flexibility for substrate and cofactor binding	[76]
Loop extensions	Reduced stability	[77]

Adapted from [22].

**Table 2:** Polar-active enzymes isolated from Antarctic and Arctic marine polar environments.

Marine Polar-Active Enzymes	Reaction	Organism Source	Origin of Sample	Applications/Potential Uses	References
**HYDROLASES: EC 3** (**Type of reaction:** Hydrolytic cleavage AB + H_2_O → AOH + BH)					
β-galactosidase	Hydrolysis of lactose into its constituent monosaccharides	*Pseudoalteromonas* sp. 22b	Alimentary tract of Antarctic krill *Thyssanoessa macrura*	Candidates for lactose removal from dairy products at low temperatures	[86,87]
β-galactosidase		*Pseudoalteromonas haloplanktis* TAE 79	Antarctic seawater		[88]
β-galactosidase		*Pseudoalteromonas haloplanktis* LMG P-19143	Antarctic seawater		[89]
β-galactosidase		*Guehomyces pullulans*	Antarctic sea sediment		[90]
β-galactosidase		*Enterobacter ludwigii*	Sediment samples of Kongsfgord, Arctic		[91]
β-galactosidase		*Alkalilactibacillus ikkense*	Ikka columns in South-West Greenland		[92]
α-Amylase	Cleavage of α-1,4-glycosidic linkages in starch molecules to generate smaller polymers of glucose units	*Pseudoalteromonas* sp. M175	Antarctic sea-ice	Detergent additive for its stain removal efficiency	[93]
α-Amylase§		*Glaciozyma antarctica* PI12	Antarctic sea-ice	Additives in processed food, in detergents for cold washing, in waste-water treatment, in bioremediation in cold climates and in molecular biology applications	[94]
α-Amylase		Bacterial strains	Sediment samples from Midtre Lovènbreen Arctic glacier		[95]
α-Amylase		*Alteromonas* sp. TAC 240B	Antarctic seawater		[96]
α-Amylase		Pseudoalteromonas haloplanktis *	Antarctic seawater		[97,98]
Xylanase	Hydrolysis of the main chain of xylan to oligosaccharides, which in turn are degraded to xylose	*Cladosporium* sp.	Antarctic marine sponges	Additives in textile and food industries, and bioremediation	[99]
Xylanase		*Flavobacterium frigidarium* sp.	Antarctic shallow-water marine sediment		[100]
Serine protease (Subtilisin)	Cleavage of peptide bonds	*Bacillus* TA39	Antarctic seawater	Additives in low-temperature food processing, food and textile industries, leather processing, detergent industry	[101,102]
Serine protease (Subtilisin)		*Bacillus* TA41	Antarctic seawater		[101,103]
Serine protease		*Colwellia* sp. NJ341	Antarctic sea-ice		[104]
Serine alkaline protease		*Shewanella* sp. Ac10u	Antarctic seawater		[105]
Acid protease		*Rhodotorula mucilaginosa* L7	Antarctic marine alga		[106]
Subtilisin-like serine protease		*Pseudoalteromonas* sp., *Marinobacter* sp., *Psychrobacter* sp., *Polaribacter* sp.	Antarctic seawater and thorax, abdomen and head of krill (*Euphausia superba* Dana)		[107]
Protease		*Pseudoalteromonas* sp. NJ276	Antarctic sea-ice		[108]
Subtilisin-like Serine proteinase		*Leucosporidium antarcticum* 171	Antarctic sub-glacial waters		[109]
Aminopeptidase		*Pseudoalteromonas haloplanktis* TAC125	Antarctic seawater		[110]
Aminopeptidase		*Colwellia psychrerythraea* 34H	Greenland continental shelf sediment samples		[111,112]
Serine peptidase		*Lysobacter* sp. A03	Penguin feathers in Antarctica		[113]
Serine peptidase		*Serratia* sp.	Coastal seawater in Northern Norway		[114]
Metalloprotease		*Pseudoalteromonas* sp. SM495	Arctic sea-ice (Canadian Basin)		[115]
Metalloprotease		*Sphingomonas paucimobilis*	Stomach of Antarctic krill, *Euphausia superba* Dana		[116]
Metalloprotease		*Psychrobacter proteolyticus* sp.	Stomach of Antarctic krill *Euphausia superba* Dana		[117]
Endopeptidase		Microbial source	Arctic marine microbial source	Candidate for molecular biology application: digestion of chromatin (ArcticZymes)	[118]
Lipase	Hydrolysis of long-chain triacylglycerol substances with the formation of an alcohol and a carboxylic acid	*Bacillus pumilus* ArcL5	Arctic seawater (Chukchi Sea)	Detergent additives used at low temperatures and biocatalysts for the biotransformation of heat-labile compounds	[119]
Lipase		*Pseudoalteromonas haloplanktis* TAC125	Antarctic seawater		[120]
Lipase		*Colwellia psychrerythraea* 34H	Arctic seawater		[121]
Lipase		*Polaromonas vacuolata*	Antarctic seawater		[122]
Lipase		*Psychrobacter* sp.	Antarctic seawater		[123,124]
Lipase		*Shewanella frigidimarina*	Antarctic seawater		[125]
Lipase		Bacterial strains	Arctic sediment samples from the snout of Midtre Lovènbreen glacier up to the convergence point with the sea		[95]
Lipase		*Psychrobacter* sp. TA144 **	Antarctic seawater		[126]
Lipase		*Psychrobacter* sp. 7195	Antarctic deep-sea sediment (Prydz Bay)		[127]
Lipase		*Moritella* sp. 2-5-10-1	Antarctic deep-sea water		[128]
Lipase		*Pseudoalteromonas* sp., *Psychrobacter* sp., *Vibrio* sp.	Antarctic seawater samples (Ross Sea)		[129]
Phytase	Hydrolysis of phytate to phosphorylated myo-inositol derivatives	*Rhodotorula mucilaginosa* JMUY14	Antarctic deep-sea sediment	Candidate for feed applications, especially in aquaculture	[130]
Esterase	Hydrolysis of simple esters, usually only triglycerides composed of fatty acids shorter than C 8	*Pseudoalteromonas arctica*	Arctic sea-ice from Spitzbergen, Norway	Additives in laundry detergents and biocatalysts for the biotransformation of labile compounds at low temperatures	[131]
Esterase		*Thalassospira* sp.	Arctic sea fan (*Paramuricea placomus*), Vestfjorden area (Northern Norway)		[132]
Esterase		*Oleispira antarctica*	Antarctic coastal waters		[73,133]
Esterase		*Pseudoalteromas haloplanktis* TAC125	Antarctic seawater		[134,135]
Esterase		*Pseudoalteromas* sp. 643A	Alimentary tract of Antarctic krill *Euphasia superba* Dana		[136]
Esterase		Marine Arctic metagenomics libraries	Arctic seawater and sediment from Barents Sea and Svalbard	Candidate for organic synthesis reactions and cheese ripening processes	[137,138]
Epoxide hydrolase	Hydrolysis of an epoxide to its corresponding vicinal diol with the addition of a water molecule to the oxirane ring	*Sphingophyxis alaskensis*	Arctic seawater	Candidate for the production of enantiopure epoxides in the pharmaceutical industry	[139]
S-formylglutathione hydrolase	Hydrolysis of S-formylglutathione to formic acid and glutathione	*Pseudoalteromonas haloplanktis* TAC125	Antarctic seawater	Candidates for chemical synthesis and industrial pharmaceutics	[140]
S-formylglutathione hydrolase		*Shewanella frigidimarina*	Antarctic marine environment		[141]
Polygalacturonase (pectin depolymerase)	Cleavage of glycosidic bonds between galacturonic acid residues	*Pseudoalteromonas haloplanktis*	Antarctic seawater	Additive in food industries, such as clarification of juice, in the process of vinification, yield and color enhancement and in the mashing of fruits	[142]
Pullulanase	Hydrolysis of α-1,6-glycosidic bonds in pullulan to produce maltotriose	*Shewanella arctica*	Seawater samples in Spitsbergen, Norway	Additive in food and biofuel industries	[143]
Invertase	Hydrolysis of the terminal non-reducing β-fructofuranoside residue in sucrose, raffinose and related β-D-fructofuranosides	*Leucosporidium antarcticum*	Antarctic seawater	Not defined (ND)	[144]
α-glucosidase	Hydrolysis of the non-reducing terminal α-glucopyranoside residues from various α-glucosides and related compounds	*Leucosporidium antarcticum*	Antarctic seawater	Additive in detergent and food industries	[144]
Cellulase	Hydrolysis of the β-1,4-D-glycosidic linkages in cellulose	*Pseudoalteromonas haloplanktis*	Antarctic seawater	Additive in detergent industry	[145]
Chitobiase	Hydrolysis of chitobiose to N-acetylglucosamine	*Arthrobacter* sp. TAD20	Antarctic sea sediments	ND	[146]
Alkaline phosphatase	Hydrolysis and transphosphorylation of a wide variety of phosphate monoesters	TAB5 strain	Antarctica^	Candidate for molecular biology application: dephosphorylation of DNA (New England Biolabs)	[147,148,149]
Alkaline phosphatase		*Shewanella* sp.	Intestine of Antarctic shellfish	Candidate for molecular biology application	[150]
Pyrophosphatase	Catalysis of the conversion of one ion of pyrophosphate to two phosphate ions	*Oleispira antarctica*	Antarctic deep sea	ND	[73]
Glycerophosphodiesterase	Catalysis of the hydrolysis of a glycerophosphodiester	*Oleispira antarctica*	Antarctic deep sea	ND	[73]
Endonuclease (Cryonase)	Cleavage of the phosphodiester bond the middle of a polynucleotide chain	*Shewanella* sp. Ac10	Antarctic seawater	Candidate for molecular biology application: digestion of all types of DNA and RNA at cold temperatures (Takara-Clontech)	[151]
Exonuclease	Cleavage of the phosphodiester bond at either the 3′ or the 5′ end	Arctic marine bacterium	Arctic marine microbial source	Candidate for molecular biology application: 3′-5′ exonuclease specific for single stranded DNA (ArcticZymes)	[152]
Ribonuclease	Hydrolysis of the phosphodiester bonds among the nucleic acid residues of RNA	*Psychrobacter* sp. ANT206	Antarctic sea-ice	Candidate for molecular biology applications	[153]
Uracil-DNA glycosylase	Hydrolysis of the N-glycosidic bond from deoxyuridine to release uracil	Antarctic marine bacterium	Antarctic marine microbial source	Candidate for molecular biology application: release of free uracil from uracil-containing single-stranded or double-stranded DNA (New England Biolabs)	[154]
**OXIDOREDUCTASES: EC 1** (**Type of reaction:** Transfer of hydrogen or oxygen or electrons between molecules AH + B → A + BH; A + O → AO; A^-^+B→A+B^-^)					
Phenylalanine hydroxylase	Catalysis of the hydroxylation of L-Phe to form tyrosine	*Colwellia psychrerythraea* 34H	Arctic marine sediments	ND	[76]
Alcohol dehydrogenase	Catalysis of the interconversion of alcohols to their corresponding carbonyl compounds	*Moraxella* sp. TAE123	Antarctic seawater	Candidate for asymmetric synthesis	[155]
Alanine dehydrogenase	Catalysis of reversible deamination of L-alanine to pyruvate	*Shewanella* sp. Ac10u, *Carnobacterium* sp. St2	Antarctic seawater	Candidate for enantioselective production of optically active amino acids	[156]
Leucine dehydrogenase	Catalysis of reversible L-leucine and other branched chain L-amino acids deamination reaction to the corresponding α-keto acid	*Pseudoalteromonas* sp. ANT178	Antarctic sea-ice	Candidate for medical and pharmaceutical industry applications	[157]
Malate dehydrogenase	Catalysis of reversible oxidation of malate to oxalacetate	*Flavobacterium frigidimaris* KUC-1	Antarctic seawater	Candidate for detection and production of malate under cold conditions	[158]
Isocitrate dehydrogenase	Catalysis of decarboxylation of isocitrate to α-ketoglutarate and CO_2_	*Desulfotalea psychrophila*	Arctic marine sediments	ND	[159]
L-threonine dehydrogenase	Catalysis of dehydrogenation at the β-carbon (C3) position of L-threonine	*Flavobacterium frigidimaris* KUC-1 ***	Antarctic seawater	ND	[160]
Superoxide dismutase	Catalysis of the dismutation of superoxide anion radicals into molecular oxygen and hydrogen peroxide	*Pseudoalteromonas haloplanktis*	Antarctic seawater	Candidates for applications in agriculture, cosmetics, food, healthcare products and medicines	[161]
Superoxide dismutase		*Marinomonas* sp. NJ522	Antarctic sea-ice		[162]
Superoxide dismutase		*Pseudoalteromonas* sp. ANT506	Antarctic sea-ice		[163]
Superoxide dismutase		*Psychromonas arctica*	Arctic sea-ice and sea-water samples		[164]
Superoxide dismutase		*Rhodotorula mucilaginosa* AN5	Antarctic sea-ice		[165]
Catalase	Catalysis of degradation of hydrogen peroxide into water and molecular oxygen	*Bacillus* sp. N2a	Antarctic seawater	Candidate for textile and cosmetic industries	[166,167]
Glutathione reductase	Catalysis of the reduction of oxidized glutathione to produce reduced glutathione	*Colwellia psychrerythraea*	Antarctic seawater	Candidate as an antioxidant enzyme in heterologous systems	[168]
Glutathione peroxidase	Catalysis of the reduction of hydrogen peroxide and other organic peroxides	*Pseudoalteromonas* sp. ANT506	Antarctic sea-ice	ND	[169]
Thioredoxin reductase	Catalysis of the reduction of thioredoxin	*Pseudoalteromonas haloplanktis* TAC125	Antarctic seawater	ND	[170]
Glutaredoxin	Catalysis of the reduction of protein disulfides in glutathione-dependent reactions	*Pseudoalteromonas* sp. AN178	Antarctic sea-ice	ND	[171]
Peroxiredoxin	Catalysis of the reduction of hydrogen peroxide, peroxynitrite and a wide range of organic hydroperoxides	*Psychrobacter* sp. ANT206	Antarctic sea-ice	Candidate for food and pharmaceutical industries	[172]
Dihydroorotate oxidase	Catalysis of the stereospecific oxidation of (S)-dihydroorotate to orotate	*Oleispira antarctica*	Antarctic deep sea	ND	[73]
**TRANSFERASES: EC 2** (**Type of reaction:** Transfer of groups of atoms AB + C → A + BC)					
Aspartate aminotransferase	Catalysis of transamination reaction of L-aspartate and α-ketoglutarate into the corresponding oxaloacetate and L-glutamate	*Pseudoalteromonas haloplanktis* TAC125 ****	Antarctic seawater	ND	[173]
Glutathione S-transferase	Catalysis of conjugation of reduced glutathione with various electrophilic compounds and ROS	*Pseudoalteromonas* sp. ANT506	Antarctic sea-ice	ND	[174]
Hydroxymethyl-transferase	Catalysis of reversible conversion of L-serine and tetrahydropteroylglutamate to glycine and 5,10-methylenetetrahydropteroylglutamate. Cleavage of many 3-hydroxyamino acids and decarboxylation of aminomalonate	*Psychromonas ingrahamii*	Arctic polar sea-ice	Candidate as a pharmaceutical, agrochemicals and food additive	[175]
**LIGASES: EC 6** (**Type of reaction:** Covalent joining of two molecules coupled with the hydrolysis of an energy rich bond in ATP or similar triphosphates A + B+ ATP → AB + ADP + Pi)					
Glutathione synthetase	Catalysis of formation of glutathione from L-γ-glutamylcysteine and glycine	*Pseudoalteromonas haloplanktis*	Antarctic seawater	ND	[176]
DNA ligase	Catalysis of the formation of a phosphodiester bond between adjacent 5′-phosphoryl and 3′-hydroxyl groups in double stranded DNA	*P. haloplanktis* TAE 72	Antarctic seawater	Candidate for applications in molecular biology	[177]
**LYASES: EC 4** (**Type of reaction:** Cleavage of C-C, C-O, C-S, C-N or other bonds by other means than by hydrolysis or oxidation RCOCOOH → RCOH + CO_2_)					
γ-carbonic anhydrase	Catalysis of CO_2_ hydration to bicarbonate and protons	*Colwellia psychrerythraea*	Antarctic cold ice sediments	Candidates for biomedical applications	[178]
γ-carbonic anhydrase		*Pseudoalteromonas haloplanktis*	Antarctic seawater		[179,180]
Pectate lyase	Cleavage of the α-1,4 glycosidic bonds of polygalacturonic acid into simple sugars	*Pseudoalteromonas haloplanktis* ANT/505	Antarctic sea-ice	Candidate for detergent industry	[167,181]
Acid decarboxylase	Catalysis of decarboxylation of 3-octaprenyl-4-hydroxybenzoate to produce 2-polyprenylphenol	*Colwellia psychrerythraea* 34H	Arctic marine sediments	ND	[182,183]
**ISOMERASES: EC 5** (**Type of reaction:** Transfer of group from one position to another within one molecule AB → BA)					
Sedoheptulose 7- phosphate isomerase	Catalysis of the conversion of sedoheptulose 7-phosphate to D-glycero-D-mannoheptose 7-phosphate	*Colwellia psychrerythraea 34H*	Arctic marine sediments	Candidate for biocatalysis under low water conditions	[184]
Triose phosphate isomerase§	Catalysis of the isomerization of dihydroxyacetone phosphate to D-glyceraldehyde 3-phosphate	*Pseudomonas* sp. π9	Antarctic sea-ice		[185]
Triose phosphate isomerase		*Moraxella* sp. TA137	Intestine of Antarctic fish		[186]

* previously known as *Alteromonas haloplanktis* [187], ** previously known as *Moraxella* TA144 [188], *** formerly known as *Cytophaga sp.* KUC-1 [189], **** formerly known as *Moraxella* TAC 125 [190]. ^it was isolated from Dumont d’Urville Antarctic Station but it was not possible to ascertain the marine origin. § only characterization of genes.
